# Supplementary material for: Longitudinal associations between hearing aid usage and cognition in community-dwelling Japanese older adults with moderate hearing loss
Source: PLoS One. 2021 Oct 13;16(10):e0258520. doi: 10.1371/journal.pone.0258520 (PMC8513843; doi:10.1371/journal.pone.0258520)
Supplement: S1 Table — Adjusted for age, sex, PTABHE, education year, history of hypertension, history of dyslipidemia, history of diabetes, history of ischemic heart disease, history of stroke, smoking status, obesity, marital status, income, depression, and occupation at baseline. (DOCX) [file pone.0258520.s001.docx]

S1 Table. Results of general linear mixed model for WAIS-R-SF scores excluding data from Times 6-8; 7-8 and 8.

|  |  | Information | | | Similarities | | | Picture completion | | | Digit symbol substitution | | |
| --- | --- | --- | --- | --- | --- | --- | --- | --- | --- | --- | --- | --- | --- |
|  |  | Estimate | SE | p-value | Estimate | SE | p-value | Estimate | SE | p-value | Estimate | SE | p-value |
| Time 1-Time 5 | Time | -0.157 | 0.041 | 0.0001 | -0.036 | 0.051 | 0,486 | 0.051 | 0.04 | 0.328 | -0.570 | 0.079 | <.0001 |
|  | Usage of hearing aid | -0.043 | 0.547 | 0.938 | 0.492 | 0.531 | 0.355 | 1.630 | 0.401 | <.0001 | 2.044 | 1.091 | 0.062 |
|  | Time × Usage of hearing aid | 0.090 | 0.140 | 0.140 | -0.044 | 0.076 | 0.563 | -0.044 | 0.059 | 0.463 | -0.114 | 0.120 | 0.341 |
| Time 1-Time 6 | Time | -0.161 | 0.038 | <.0001 | -0.040 | 0.045 | 0.024 | 0.065 | 0.038 | 0.137 | -0.575 | 0.079 | <.0001 |
|  | Usage of hearing aid | -0.063 | 0.545 | 0.904 | 0.533 | 0.525 | 0,310 | 1.628 | 0.399 | <.0001 | 2.207 | 1.095 | 0.044 |
|  | Time × Usage of hearing aid | 0.100 | 0.054 | 0.065 | -0.066 | 0.065 | 0.307 | -0.048 | 0.055 | 0.378 | -0.171 | 0.118 | 0.146 |
| Time 1-Time 7 | Time | -0.167 | 0.035 | <.0001 | -0.057 | 0.045 | 0.013 | 0.057 | 0.034 | 0.100 | -0.581 | 0.076 | <.0001 |
|  | Usage of hearing aid | -0.089 | 0.545 | 0.870 | 0.504 | 0.522 | 0.335 | 1.602 | 0.395 | <.0001 | 2.243 | 1.093 | 0.041 |
|  | Time × Usage of hearing aid | 0.111 | 0.05 | 0.024 | -0.046 | 0.064 | 0.473 | -0.033 | 0.048 | 0.498 | -0.182 | 0.111 | 0.102 |

Adjusted for age, sex, PTABHE, number of years of education, history of hypertension, history of dyslipidemia, history of diabetes, history of ischemic heart disease, history of stroke, smoking status, obesity, marital status, income, and occupation at baseline.
