# Supplementary material for: Longitudinal associations between hearing aid usage and cognition in community-dwelling Japanese older adults with moderate hearing loss
Source: PLoS One. 2021 Oct 13;16(10):e0258520. doi: 10.1371/journal.pone.0258520 (PMC8513843; doi:10.1371/journal.pone.0258520)
Supplement: Attachment — Submitted filename: response to reviewers.docx [file pone.0258520.s002.docx]

**Response to Reviewers**

Our incorporation of the Reviewer comments is as follows:

The changed part in the manuscript was shown in red color.

**To reviewer #1:** This epidemiologic study focused on the protective effect of hearing aids in older adults. The experiment itself is carried out with careful treatments, but the reviewer thinks the sample size is too small.

→Thank you for reviewing our manuscript. As you pointed out, the sample size was not large. However, NILS-LSA is one of the largest cohort studies in Japan that assessed pure-tone audiometry, and we tried to increase the sample size by collecting and reconstructing the samples with moderate hearing loss from all participants. We mentioned this limitation in discussion. (Line 107-110, 345-350)

Methods

Why were the participants under 60 years old excluded?

Please explain the reason. Usually, the definition of old age is over 65 years old.

→In Japan, people over 60 have been regarded as elderly and retired. Of course, the current situation is that they are being reviewed to make them over 65. Also, the NILS-LSA was designed to have the same number of participants in each decade (40s, 50s, 60s, 70s). Thus, we did not change the definition of old age as over 65 years old in accordance with our previous study.

Table 1

This table was not informative. Please create a flow-chart.

The number of participants on each group should be shown.

→We added a flow-chart (Fig 1). We also added the number of HA users to Table 1 as the reviewer#2 suggested.

Table 1

The follow-up rates were under 20% from “Time 6”. How were the results changed, if the analysis was performed until “Time5”?

→We performed additional analyses excluding Time 6-8, and added these results as S1 Table. (Line 200, 201, 261-264)

Methods

Were the participants with otological diseases, like otitis media excluded? Please explain it.

→We did not exclude the participants who had the history of ontological diseases because there were 50 participants of 407 whose data was missing. We added the explanation and discussed it. (Line 114, 115, 345-350)

Methods

The reviewer thought that the covariates were not enough in this study. The previous report which the authors published on Auris Nasus Larynx included more covariates than this study. Please include more covariates and re-calculate the data. Obesity, history of hyperlipidemia, alcohol intake and history of cardiovascular disease, like stroke and ischemic heart disease are essential covariates, when the cognitive functions are analyzed.

→Choice of covariates is difficult problem. We added obesity, history of hyperlipidemia, and history of ischemic heart disease as suggested. We did not add alcohol intake because there were 48 participants of 407 who lack an answer for the questionnaire about alcohol intake. Again, we discussed this limitation. (Line 345-350)

Results

Why were the picture completion scores higher than baseline on both groups? Are the picture completion tests not useful tests on evaluating cognitive function? Please explain it.

→It has been suggested that the Picture completion might be resilient to the impact of brain damage (Kaufman AS & Lichtenberger EO, Assessing adolescent and adult intelligence. 3^rd^ ed. Hoboken, NJ: John Wiley & Sons, 2006), and to hold up with age better than most other performance tests (Wechsler D. The measurement of adult intelligence. 3^rd^ et. Baltimore, OH: The Williams & Wilkins Company, 1944). Thus, we thought scores of Picture completion did not decrease after adjusting for covariates such as practice effects. We discussed it in previous study (Niahita Y, et al. Does high educational level protect against intellectual decline in older adults? : A 10-year longitudinal study. Jpn Psychol Res 2013;55:378-389). We added the reference and mentioned it. (Line 246, 247, 289-294)

Results

Only one model was shown on Table 3. Please create more than two models.

→We create two models, model 1 is adjusted with covariates same as our previous study (Uchida Y, et al. The longitudinal impact of hearing impairment on cognition differs according to cognitive domain. Front Aging Neurosci 2016;8:201), and model 2 is adjusted with covariates model 1 + depression, income, obesity, and history of dyslipidemia.

Discussion from 297 to 308

This paragraph should be stated at method section.

→We moved this paragraph to method section. (Line 154-165)

Abbreviations about hearing aids were incorrect like “Has” at some points. Please correct them to “HA”.

→We corrected as suggested.

**To reviewer #2**: This is a well-conducted study of the association of hearing aid usage with longitudinal cognitive function among 407 older adults with moderate hearing impairment. Given the prevalence of hearing impairment, and prevalence of cognitive decline with age, this is an important and relatively under-studied area. This study represents an important contribution to the literature. Strengths include the relatively large number of participants; objective measures of hearing thresholds; control for many important potentially confounding factors; longitudinal measures, and use of general linear mixed effects regression models. Limitations are adequately noted. However, I have several suggestions for improvement:

→We deeply appreciate your kind comments.

Title: Since this is an observational study that describes the association between hearing aid usage and cognitive decline it is important to avoid language the implies causation. Thus the word “Effects” should be changed to “Associations” in the title and throughout the text.

→We changed as suggested.

For the full implication of the results to be appreciated from the abstract, it is important to state that slopes did not differ between HA users and non-users for the Similarities, Picture Completion and Digit Symbol Substitution tests.

→We added a sentence as suggested. (Line 44, 45)

Line 49 include “is”: The global population of older adults is expected to reach 1.4 billion by 2030 and 2.1 billion by 2050.

→We changed as suggested. (Line 51)

Line 88: avoid causal language such as “HAs use influenced changes…” since differences between HA users and non-users may arise from uncontrolled confounding.

→We changed as suggested. (Line 89, 90)

In a few places in the manuscript, hearing aid use is sometimes abbreviated as “ha” or “has” – rather than HA or HAs.

→We corrected as suggested.

It would be helpful if Table 1 was stratified by hearing aid use and non-hearing aid use to allow the reader to appreciate the difference in participation/follow-up time between these two groups.

→We added the number of HA users to Table 1 as suggested.

It would be helpful to report on the average time interval between baseline and report of HA use, as well as the range of that interval. This is important to appreciate how much time may have passed between the onset of hearing impairment and beginning of use of hearing aids, which could affect associations with cognitive function.

→There were 66 participants who had moderate hearing loss and use HA from the first participation, and we could not know when they began to use HA. So, we added the average time interval between baseline and report of HA use in remaining 62 participants. (Line 121-124)

Line 312-313. It is unclear why being a local resident would affect whether participants considered PSAPs as HAs. It is not clear whether the authors are implying that participants may have indicated HA use when they were not using HA, but were using PSAPs? "In addition, personal sound amplification products (PSAPs) may be treated as HAs, because the participants were local residents so they may not be able to distinguish HA from PSAPs”.

→Opposite to the United States, HA has been available to the consumer through Internet or retail outlets, for example, eyeglass shop, in Japan for a long time. Also, pre-purchase hearing evaluation is not necessary to buy HA. So, people sometimes mistake PSAPs as HA in Japan. We added a sentence explaining the above. (Line 337-341)

Conclusion: As with the abstract, it is important to note the implications of the non-significant findings as well. That is, these results shows that HA usage is not associated with reduction in rate of cognitive decline in some tasks that show significantly higher rates of decline among individuals with hearing impairment relative to those without hearing impairment.

→We added a sentence as suggested. (Line 355, 356)

We corrected some errors, and added one recent study (Line 285, 286, Reference number 33).
