# Supplementary material for: Longitudinal associations between hearing aid usage and cognition in community-dwelling Japanese older adults with moderate hearing loss
Source: PLoS One. 2021 Oct 13;16(10):e0258520. doi: 10.1371/journal.pone.0258520 (PMC8513843; doi:10.1371/journal.pone.0258520)
Supplement: Attachment — Submitted filename: response to reviewers0926.docx [file pone.0258520.s003.docx]

**Response to Reviewers**

Our incorporation of the Reviewer comments is as follows:

The changed part in the manuscript was shown in red color.

To Reviewer #1:

The reviewer thinks authors properly responded to the reviewers' comments and precisely revised the manuscript.

→Thanks to your kind review, our manuscript is better.

To Reviewer #2:

The authors have been responsive to prior reviewers and have improved the manuscript. I note below a few minor suggestions to improve clarity:

→Thank you for reviewing our manuscript.

Abstract line 40 : remove “therefore”

→We corrected as suggested.

Abstract lines 41 – 43: The following sentence is unclear: “The model-predicted 12-year slope

with centralizing age indicated that the Information score showed no significant decline in the participants using HA, although this tolerance was not found in the participants not using HA.” Do the authors mean that non-HA users showed significant decline in Information scores over the 12 year follow-up period whereas HA users did not? Since the slope in the HA group approached significance, I believe a better interpretation would be that the non HA users showed greater decline over time on Information scores than did HA users.

→We corrected as suggested.

Results, pg 17, line 242 change were to are: “are shown in Fig 2”

→We corrected the sentence as suggested.

Results, pg 18, line 261 change were to are: “are shown in S1 Table. Also, please change the table title to be more informative. Eg. “Results of general linear mixed model for WAIS-R-SF scores excluding data from Times 6-8; 7-8 and 8”

→We corrected the sentence and changed the title as suggested.

Results Line 246 – 247. This sentence is unclear: “The Picture completion scores did not decrease especially in the non-users, however, there was no significant difference in the rate of change between two groups”. This would be better expressed as “There were no significant changes in Picture completion scores over time in either the HA or non HA users.”

→We corrected the sentence as suggested.

Table 3: I am not sure it is necessary to show the results from model 1 and model 2. There was no a priori reason for assessing differences between these models, although I understand that additional covariates were added in response to review. However, it would be more meaningful to show results of an unadjusted (or minimally adjusted model – including eg. age, sex, education) and the model with all the covariates (fully adjusted model).

→We changed the model 1 as minimally adjusted model. The sentences of results (Line 221-231) were also changed.
